# Exploring the relationship between age and prognosis in glioma: rethinking current age stratification

**DOI:** 10.1186/s12883-022-02879-9

**Published:** 2022-09-15

**Authors:** Zetian Jia, Xiaohui Li, Yaqi Yan, Xuxuan Shen, Jiuxin Wang, He Yang, Shuo Liu, Chengxi Han, Yuhua Hu

**Affiliations:** 1 Department of Neurosurgery, The First Hospital of Handan of Hebei Province, Handan, 056000 People’s Republic of China; 2 School of Life Science and Engineering, Handan University, 056000 Handan, People’s Republic of China; 3 Department of Food Science and Technology, Graduate School, Woosuk University, 55338 Wanju-gun, Korea; 4 Department of Cardiology, The First Hospital of Handan of Hebei Province, Handan, 056000 People’s Republic of China; 5 Department of Neurosurgery, Affiliated Dongyang Hospital of Wenzhou Medical University, Dongyang, Zhejiang, 322100 China; 6 Department of Neurosurgery, The Second Hospital of Hebei Medical University, Shijiazhuang, 050000 People’s Republic of China

**Keywords:** Age, Akaike information criterion, Brain tumors, Central nervous system, Glioblastoma multiforme, Glioma, High grade gliomas, Low grade gliomas, Restricted cubic spline, SEER

## Abstract

**Background:**

The age of glioma plays a unique role in prognosis. We hypothesized that age is not positively correlated with survival prognosis and explored its exact relationship.

**Methods:**

Glioma was identified from the SEER database (between 2000 and 2018). A multivariate Cox proportional regression model and restricted cubic spline (RCS) plot were used to assess the relationship between age and prognosis.

**Results:**

A total of 66465 patients with glioma were included. Hazard ratios (HR) for ten-year by age: 0–9 years, HR 1.06 (0.93–1.20); 10–19 years: reference; 20–29 years, HR 0.90 (0.82–1.00); 30–39 years, HR 1.14 (1.04–1.25); 40–49 years, HR 2.09 (1.91–2.28); 50–59 years, HR 3.48 (3.19–3.79); 60–69 years, HR 4.91 (4.51–5.35);70–79 years, HR 7.95 (7.29–8.66); 80–84 years, HR 12.85 (11.74–14.06). After adjusting for covariates, the prognosis was not positively correlated with age. The smooth curve of RCS revealed this non-linear relationship: HR increased to 10 years first, decreased to 23 years, reached its lowest point, and became J-shaped.

**Conclusion:**

The relationship between age and glioma prognosis is non-linear. These results challenge the applicability of current age groupings for gliomas and advocate the consideration of individualized treatment guided by precise age.

**Supplementary Information:**

The online version contains supplementary material available at 10.1186/s12883-022-02879-9.

## Background

Glioma is the most common primary tumor of the intracranial central nervous system (CNS), accounting for approximately 30% of all CNS tumors and 80% of malignant intracranial tumors [1]. Glioma incidence is rare in the United States; however, it has a high mortality rate due to the specific site and other tumor characteristics. The five-year survival rate for primary brain malignancies is approximately 33% [2]. Particularly for patients with glioblastoma multiforme (GBM), median survival is only a year, even after standard treatment [3]. Current management and treatment models for gliomas are based on accurately estimated survival data, which rely on large clinical follow-up series studies. The survival of patients with glioma has increased over the past 30 years as molecular diagnostics and treatments have improved. At the same time, data on the demographic characteristics of patients with glioma based on large sample sizes have the unique advantage of demonstrating the impact of these developments on the entire population and are important for the outcome of the study.

Glioma incidence is not limited by age; however, it changes with age [4]. More than one study has shown that the age of patients with glioma is an important independent prognostic factor. Despite the same histological diagnosis, the prognosis of gliomas varies greatly by age [5, 6]. The current selection of age ranges associated with glioma patient outcomes is not entirely consistent. In addition, arbitrarily converting a continuous variable, such as age, into a categorical variable may lose some important data and lead to biased research conclusions. On the other hand, due to the different histological glioma subtypes, the prognostic effect of age on the population is controversial [7–9]. In a population-based cohort study of 1,502 gliomas, Lin et al. classified gliomas into children (<15 years), young adults (15–47 years), middle-aged adults (48–63 years), and older adults (64 years or older) and suggested that this grouping could be used to assess glioblastoma risk [10]. However, this age classification method is not currently used. Another study classified patients with glioma into old and young groups, using the age of 60 as the segmentation point [8]. The latest edition of the 2021 WHO Classification of the Central nervous system discusses childhood and adult gliomas separately [11]. High-grade gliomas, such as mesenchymal astrocytoma (grade III) and GBM (grade IV), are usually characterized by a high degree of malignancy, rapid progression, and poor prognoses [12]. The transformation of continuous variables, such as age, into dichotomous or multifractal variables is conducive to the individualized and stratified management of glioma; nevertheless, details of the impact of continuous variables on prognosis may be neglected simultaneously. Studies have failed to attain consensus on the effect of age on glioma staging. Notably, previous studies have assumed that there is an exact point at which glioma age and prognosis are associated. However, the results obtained by the prediction model do not prove the superiority of the previous hypothesis. In addition, in previous population-based glioma studies, the limitation of sample size and lack of adjustment of covariates led to the unclear relationship between age and glioma prognosis.

We attempted to explore the relationship between age at diagnosis and the prognosis of patients with glioma without making assumptions or human bias. We used statistical methods to construct a model to objectively determine whether there is an association between age and prognosis in patients with glioma.

## Methods

### Patients’ data

The clinical data for all the patients came from the Surveillance, Epidemiology, and End Results (SEER) registry's public database, which collected data on patients diagnosed with cancer from 18 registries (San Francisco-Oakland, Connecticut, Detroit, Hawaii, Iowa, New Mexico, Seattle, Utah, Atlanta, San Jose-Monterey, Los Angeles, Alaska Natives, Rural Georgia, California, Kentucky, Louisiana, New Jersey, and Greater Georgia) between 2000 and 2018 [13]. The SEER program included population-based cancer-related data (such as incidence, survival, demographics, primary tumor site, and histological diagnosis) covering approximately 28% of the U.S. population, including central nervous system tumors diagnosed in the U.S. [13]. The study was conducted in accordance with the Declaration of Helsinki.

The primary site codes of patients with glioma included based on the International Classification Diseases for Oncology, third edition (ICD-O-3) were: supratentorial (C71.0-71.4), infratentorial (C71.6 and C71.7), [14] and overlapping lesions (C71.8). Histological ICD-O-3 of glioma: diffuse astrocytoma, 9400; anaplastic astrocytoma, 9401; glioblastoma, 9440, 9441, and 9442; oligodendroglioma, 9450 [15].

We excluded patients aged 85 years and older diagnosed with glioma due to incomplete age information in the database. The diagnosis of glioma in adults and children is considered to have different disease characteristics; however, we included children in this analysis due to the consistent histological diagnosis [16]. Moreover, based on SEER database limitations, there is no molecular disease stratification, including molecular diagnosis [16]. The study population included children (0–19 years), young adults (20–39 years), adults (40–64 years), and elderly patients (65–84 years) [5, 16]. Additionally, we discussed the effect of age on glioma prognosis using age as a categorical variable in different stratification methods (Stratified each group for ten years as described above). Racial information on patients with glioma was classified as white, black, and others. The patient's sex information (female or male) was extracted from the database. To further explore the potential changes in diagnosis age on glioma prognosis, the initial diagnosis ages were defined as three time periods: 2000–2006, 2007–2012, and 2013–2018. Patients with insufficient information were excluded from the study. Finally, the sample size of patients with glioma included was 66465.

### Statistical analyses

We used Cox proportional hazards regression models with a follow-up timescale to estimate prognosis and calculate 95% confidence intervals for patients with glioma. Age was initially converted into the appropriate categorical variable according to the method described above (for comparison with the work based on our study) [5, 16]. We stratified the ages by ten years: (0–9, 10–19, 20–29, 30–39, 40–49, 50–59, 60–69, 70–79, and 80–84). The optimal subset was used to filter the independent variables of the optimal model. In addition, we constructed a multivariate model incorporating all independent variables (adjusted for age, sex, race, age at diagnosis, histology, and primary site). After applying restricted cubic spline (RCS) to construct different models, the association between glioma diagnosis age and risk ratio was visualized and compared with Akaike information criterion (AIC) models. The optimal RCS model was internally verified using the bootstrap method with 500 repeated samples to confirm the robustness of the model. In addition, 30% of the population (19,937 samples) were randomly selected as the internal validation data set to draw the RCS curve.

The RCS plot was made with age as a continuous variable. The spline knots were set at 0, 10, 19, 39, 64, and 84 years. Furthermore, flexible modeling assessed the relationship between age and glioma prognosis. In the spline model, an optimal subset was also used to screen variables, and then the potential linear or non-linear relationship between age and glioma prognosis was tested using adjusted independent variables [17]. Two inflection points in glioma outcomes were observed at ages 10 and 23 during RCS construction. The relationship between age and prognosis was roughly log-linear between 0–10 years and 11–23 years, and a linear model was constructed to calculate the proportion of increased risk per standard deviation of age. For people older than 23 years, age was not linearly correlated with prognosis; however, we used a linear model to estimate the risk ratio for each standard deviation increase in age.

In addition, we conducted a stratified analysis of the population based on the optimal subset regression model screening results to determine whether the relationship between age and glioma prognosis depends on histology, age at diagnosis, and tumor location. All the statistical tests considered significant were P<0.05. Clinical data of glioma patients were obtained using SEER* Stat 8.3.9 software. Statistical calculations and plots were performed using R software 4.0.5 (https://www.r-project.org).

## Results

### Patient characteristics

Our study included 66,465 patients with glioma. Table 1 presents the baseline characteristics of the study population by age group. The patients with glioma were mainly adults and the elderly, accounting for 45.9% and 40.2% of the total population, respectively. The number of male patients with glioma was more than the females in all age groups. In addition, glioblastoma accounts for approximately one-third of the population in children and young adults. However, the prevalence of glioblastoma in the adult and elderly populations was 77.49% and 87.9%, respectively. Supratentorial glioma remains the most common site in all populations.

**Table 1:** Characteristics of the glioma study population

Characteristics	Overall *N*=66465	Children (Birth to 19 years); *N*=2066	Young adults (20-39 years); *N*=7158	Adults (40-64 years); *N*=30495	Elderly (65-84 years); *N*=26746
Follow-up time (years)					
Mean (SD)	2.0 (3.20)	5.5 (5.67)	5.0 (4.81)	2.0 (2.99)	0.8 (1.31)
Median (IQR)	0.8 (0.25-1.92)	2.7 (0.92-9.56)	3.3 (1.25-7.58)	1.1 (0.42-2.08)	0.3 (0.17-0.92)
Gender (%)					
Female	28077 (42.24)	967 (46.81)	2947 (41.17)	12291 (40.30)	11872 (44.39)
Male	38388 (57.76)	1099 (53.19)	4211 (58.83)	18204 (59.70)	14874 (55.61)
Year diagnosed (%)					
2000-2006	22148 (33.32)	749 (36.25)	2615 (36.53)	10143 (33.26)	8641 (32.31)
2007-2012	20964 (31.54)	669 (32.38)	2124 (29.67)	9922 (32.54)	8249 (30.84)
2013-2018	23353 (35.14)	648 (31.36)	2419 (33.79)	10430 (34.20)	9856 (36.85)
Race (%)					
Black	4008 (6.03)	255 (12.34)	497 (6.94)	1989 (6.52)	1267 (4.74)
White	58747 (88.39)	1604 (77.64)	6024 (84.16)	26844 (88.03)	24275 (90.76)
Other	3710 (5.58)	207 (10.02)	637 (8.90)	1662 (5.45)	1204 (4.50)
Histology (%)					
Diffuse astrocytoma	5983 (9.00)	808 (39.11)	1700 (23.75)	2159 (7.08)	1316 (4.92)
Anaplastic astrocytoma	6058 (9.11)	377 (18.25)	1539 (21.50)	2596 (8.51)	1546 (5.78)
Glioblastoma	50128 (75.42)	623 (30.15)	2361 (32.98)	23632 (77.49)	23512 (87.91)
Oligodendroglioma	4296 (6.46)	258 (12.49)	1558 (21.77)	2108 (6.91)	372 (1.39)
Location (%)					
Infratentorial	1600 (2.41)	478 (23.14)	327 (4.57)	487 (1.60)	308 (1.15)
Supratentorial	50706 (76.29)	1261 (61.04)	5703 (79.67)	23635 (77.50)	20107 (75.18)
Overlapping lesion	14159 (21.30)	327 (15.83)	1128 (15.76)	6373 (20.90)	6331 (23.67)

*SD* standard deviation, *IQR* Inter Quartile Range

Patients with glioma were followed up for an average of two years, and 54019 (81.3%) deaths were recorded. Table 2 presents the relationship between glioma mortality and age classification. In the four age groups, the univariate model, optimal subset (Supplementary Fig. 1) screening variable model, and adjusted all variable model revealed a strong positive correlation between patients' ages and mortality risk ratio. In the optimal model (Model 2), the risk ratio for death was 4.56 (95% confidence interval 4.26–4.88) for the oldest group (65–84 years) compared to the lowest group (0–19 years). Model 1 (AIC=1092509), model 2 (AIC=1083190), and model 3 (AIC=1083110). The AIC of model 2 with three variables did not significantly differ from that of model 3 with five variables.

**Table 2:** Hazard ratio (95% CI) of mortality in patients with glioma

		Model 1		Model 2		Model 3	
Age	Mortality	HR (95% CI)	*P*-value	HR (95% CI)	*P*-value	HR (95% CI)	*P*-value
Four groups of age							
Birth to 19 years	46.7%	1 (reference)		1 (reference)		1 (reference)	
20-39 years	48.3%	1.02 (0.95 to 1.1)	0.557	1.03 (0.96 to 1.11)	0.384	1.03 (0.96 to 1.11)	0.399
40-64 years	81.2%	3.07 (2.88 to 3.27)	<0.001	2.24 (2.09 to 2.40)	<0.001	2.23 (2.09 to 2.39)	<0.001
65-84 years	92.8%	6.89 (6.45 to 7.35)	<0.001	4.56 (4.26 to 4.88)	<0.001	4.56 (4.26 to 4.88)	<0.001
Per 10 years of age							
0-9 years	47.0%	1.06 (0.93 to 1.20)	0.398	1.00 (0.88 to 1.13)	0.941	1.00 (0.88 to 1.13)	0.966
10-19 years	46.2%	1 (reference)		1 (reference)		1 (reference)	
20-29 years	43.7%	0.90 (0.82 to 1.00)	0.050	0.91 (0.82 to 1.00)	0.058	0.91 (0.82 to 1.00)	0.060
30-39 years	51.0%	1.14 (1.04 to 1.25)	0.005	1.12 (1.02 to 1.23)	0.018	1.12 (1.02 to 1.23)	0.018
40-49 years	70.9%	2.09 (1.91 to 2.28)	<0.001	1.71 (1.56 to 1.87)	<0.001	1.70 (1.56 to 1.86)	<0.001
50-59 years	83.4%	3.48 (3.19 to 3.79)	<0.001	2.41 (2.21 to 2.63)	<0.001	2.41 (2.21 to 2.63)	<0.001
60-69 years	88.4%	4.91 (4.51 to 5.35)	<0.001	3.27 (3.00 to 3.57)	<0.001	3.27 (3.00 to 3.57)	<0.001
70-79 years	93.4%	7.95 (7.29 to 8.66)	<0.001	5.18 (4.75 to 5.66)	<0.001	5.19 (4.76 to 5.67)	<0.001
80-84 years	97.0%	12.85 (11.74 to 14.06)	<0.001	8.44 (7.70 to 9.25)	<0.001	8.48 (7.74 to 9.29)	<0.001

*HR* Hazard ratio, *CI* Confidence interval. Model 1: Unadjusted covariates; Model 2: Adjusted for year (2000-2006,2007-2012 or 2013-2018), histology (diffuse astrocytoma, anaplastic astrocytoma, glioblastoma or oligodendroglioma) and location (infratentorial, supratentorial or overlapping lesion) based on optimal full subset regression. Model 3: Adjusted for year (2000-2006,2007-2012 or 2013-2018), histology (diffuse astrocytoma, anaplastic astrocytoma, glioblastoma or oligodendroglioma), location (infratentorial, supratentorial or overlapping lesion), race (black, white or other) and gender (female or male)

### Relationship between age and prognosis of glioma

**Fig. 1:**
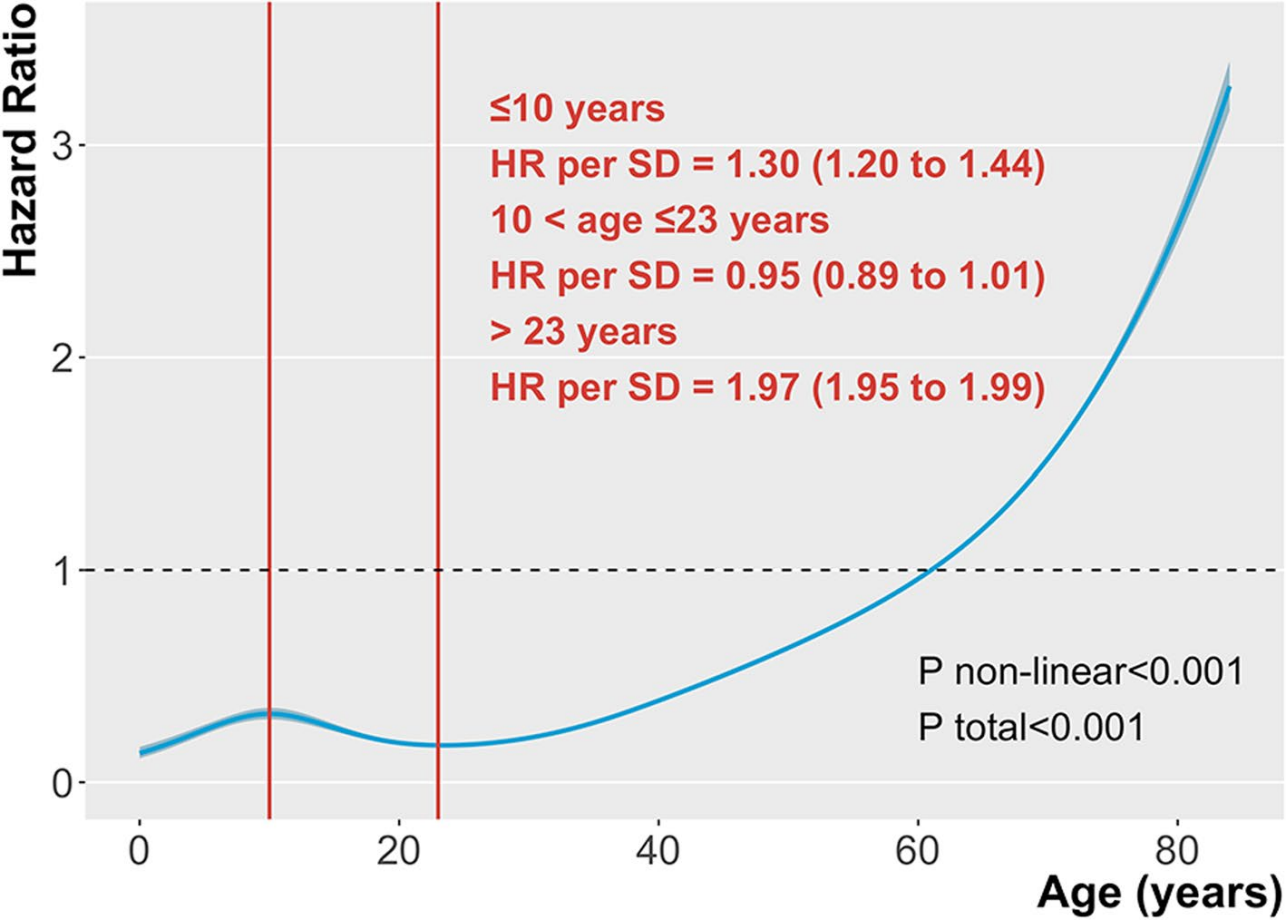
Association between age and hazard ratio of death in patients with glioma. (Unadjusted covariable)

We used the restricted cubic spline plots to model age as a continuous variable and visualized the relationship between glioma age and prognosis (Fig. 1). In the figure, two inflection points are at ages 10 and 23, with HR of approximately 0.32 (95% CI 0.30–0.35) and 0.17 (95% CI 0.17–0.18), respectively. The HR for death increased before the predicted 10 years, decreased between 10 and 23 years, and increased nearly exponentially (non-linear *P*<0.001). The HR for each standard deviation increase before age 10 was 1.30 (95% CI 1.20–1.44). In addition, the HR for each additional standard deviation of age before reaching 23 years was 0.95 (95% CI 0.89–1.01). After glioma patients aged beyond 23 years, the risk ratio per standard deviation age was 1.97 (95% CI 1.95–1.99).

**Fig. 2:**
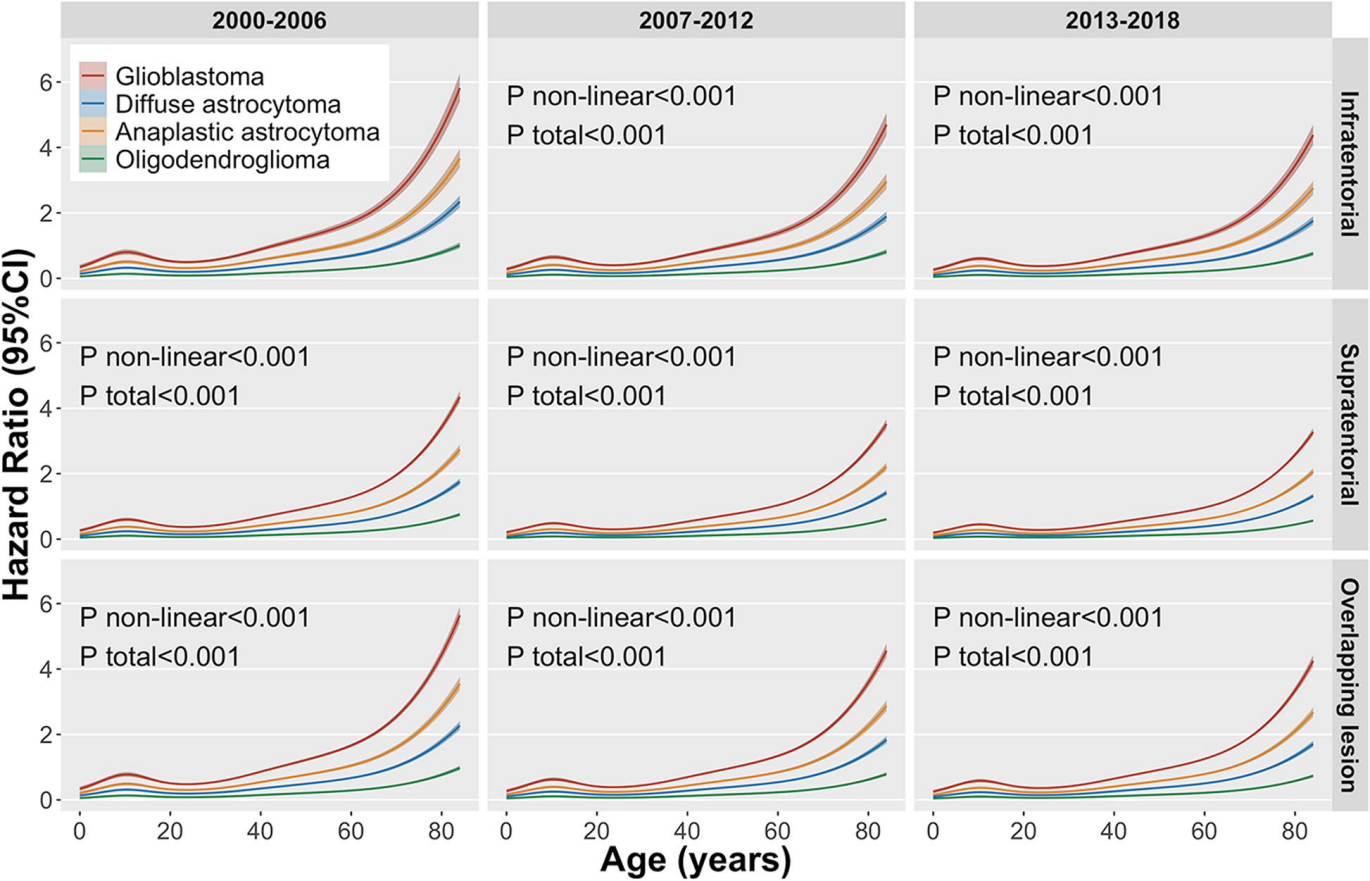
Association between age and hazard ratio of death in patients with glioma. (Adjusted histological diagnosis, diagnosis year, tumor location)

When age was grouped into groups of 10 years, we observed an approximate J-shaped association between age and glioma mortality risk ratios (Table 2 and Fig. 1). After adjusting the influence of different variables on the model, we observed that this J-shaped correlation remained (Table 2 and Fig. 2). In model 1, without adjusting variables, the risk of death in the 20–29 age group was 10% lower than that in the 10–19 age group, which was statistically critical (*P*=0.05). In model 2 and Model 3, after adjusting for other variables, the risk of death in the 20–29 age group remained the lowest among all subgroups; however, it was not significant (*P*=0.058 and *P*=0.06). In the group >29 years, the risk ratio of glioma death remained unchanged and increased rapidly. Figure 2 presents the results of the restricted cubic spline plot after adjusting for different variables. We observed that the histological diagnosis of glioma might be an important covariate affecting age and prognosis of J-shaped changes. Therefore, we performed a stratified analysis of gliomas in different case types.

### Relationship between age and glioma prognosis after histological stratification

**Fig. 3:**
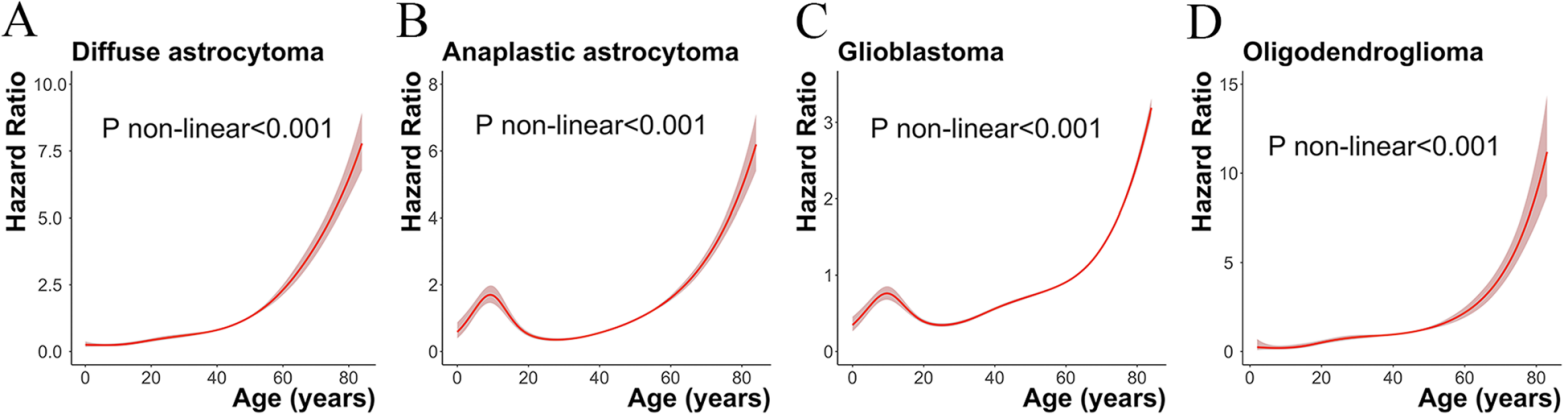
**A**: Age and mortality hazard ratio in patients with diffuse astrocytoma; **B**: Age and mortality hazard ratio in patients with anaplastic astrocytoma; C: Age and mortality hazard ratio in patients with glioblastoma; **C**: Age and mortality hazard ratio in patients with oligodendroglioma

We stratified gliomas according to different histologic types and observed that the mortality risk ratio of anaplastic astrocytoma and glioblastoma in patients aged 20–29 years remained the lowest compared to other groups (Table 3). However, this change was not significant in diffuse astrocytoma and oligodendroglioma. Restricted cubic spline plots also confirmed that the age of patients with different histological types of glioma affected prognosis differently (Fig. 3).

**Table 3:** Hazard ratio (95% CI) of mortality in patients with glioma (Based on histological stratification)

	Diffuse astrocytoma		Anaplastic astrocytoma		Glioblastoma		Oligodendroglioma	
Age	HR (95% CI)	*P*-value	HR (95% CI)	*P*-value	HR (95% CI)	*P*-value	HR (95% CI)	*P*-value
Four groups of age								
Birth to 19 years	1 (reference)		1 (reference)		1 (reference)		1 (reference)	
20-39 years	2.47 (2.08 to 2.95)	<0.001	0.46 (0.40 to 0.53)	<0.001	0.76 (0.68 to 0.84)	<0.001	3.03 (2.05 to 4.48)	<0.001
40-64 years	5.74 (4.85 to 6.79)	<0.001	1.07 (0.94 to 1.22)	0.319	1.47 (1.34 to 1.60)	<0.001	5.21 (3.55 to 7.65)	<0.001
65-84 years	16.34 (13.76 to 19.40)	<0.001	3.04 (2.66 to 3.48)	<0.001	2.85 (2.61 to 3.12)	<0.001	16.98 (11.40 to 25.29)	<0.001
Per 10 years of age								
0-9 years	1.05 (0.77 to 1.44)	0.751	1.36 (1.07 to 1.72)	0.012	1.04 (0.87 to 1.24)	0.682	1.30 (0.58 to 2.89)	0.521
10-19 years	1 (reference)		1 (reference)		1 (reference)		1 (reference)	
20-29 years	2.31 (1.80 to 2.98)	<0.001	0.47 (0.38 to 0.57)	<0.001	0.67 (0.58 to 0.77)	<0.001	2.99 (1.82 to 4.90)	<0.001
30-39 years	2.79 (2.18 to 3.57)	<0.001	0.55 (0.46 to 0.67)	<0.001	0.82 (0.72 to 0.93)	0.002	3.48 (2.16 to 5.61)	<0.001
40-49 years	3.62 (2.84 to 4.63)	<0.001	0.75 (0.63 to 0.91)	0.003	1.19 (1.06 to 1.34)	0.003	4.39 (2.73 to 7.05)	<0.001
50-59 years	7.59 (5.97 to 9.63)	<0.001	1.53 (1.28 to 1.83)	<0.001	1.50 (1.34 to 1.68)	<0.001	6.65 (4.13 to 10.70)	<0.001
60-69 years	11.25 (8.86 to 14.28)	<0.001	2.32 (1.94 to 2.78)	<0.001	2.00 (1.79 to 2.25)	<0.001	11.58 (7.15 to 18.75)	<0.001
70-79 years	19.66 (15.46 to 25.00)	<0.001	3.96 (3.30 to 4.76)	<0.001	3.16 (2.82 to 3.54)	<0.001	23.11 (14.07 to 37.98)	<0.001
80-84 years	28.65 (21.93 to 37.42)	<0.001	6.61 (5.34 to 8.18)	<0.001	5.19 (4.62 to 5.83)	<0.001	46.44 (26.56 to 81.19)	<0.001

*HR* Hazard ratio, *CI* Confidence interval. Adjusted for year (2000-2006,2007-2012 or 2013-2018), location (infratentorial, supratentorial or overlapping lesion), race (black, white or other) and gender (female or male)

## Discussion

This large retrospective cohort study of gliomas explored the relationship between patient age and prognosis. In the unadjusted covariate model analysis, the prognosis of patients with glioma increased first to 10 years and then decreased to the lowest point (23 years). After age 23, glioma prognosis increased with age in a J-shaped pattern. This relationship persisted after adjusting for clinical characteristics of patients with glioma. The RCS plot revealed that the age of glioma was not completely positively correlated with the HR, and the prognosis at age 23 seemed better than that at age 10. It seems that the age stratification for children, adolescents, and young adults should be reconsidered, challenging the applicability of current age groups for gliomas.

Published data in recent years have questioned the applicability of age grouping of patients with glioma; however, the issue remains controversial given the inconsistencies in published studies [5, 16, 18]. Chen et al. [18] analyzed clinical and follow-up data of 125 patients with high-grade gliomas (HGG) who underwent surgery and were pathologically diagnosed at a single-center medical facility between 2002 and 2012. Their study investigated the relationship between different age classification criteria and HGG prognosis. Their study reported 86 deaths in patients with HGG during a mean follow-up of 23.2 months. The authors concluded that considering age 50 as the cutoff point to divide the population into two categories is the most appropriate independent prognostic factor for patients with HGG. In another study, the authors analyzed the relationship between age and prognosis in patients with GBM at a cutoff point of 60 years [8]. The study included 35 patients surgically diagnosed with GBM at a single medical center between 2003 and 2005. The mean follow-up was 9.5 months, and the survival rate was 16% at 20 months [8]. The authors conclude that age (≥60 vs. <60 years) can be used as an independent prognostic factor in the GBM patient population but loses power in the outcome.

The two cohort analyses mentioned above also studied the influence of age on the prognosis of glioma patients; however, the results were contradictory due to different age cutoff points. The prognosis of gliomas varies with different histological diagnoses, especially for high-grade gliomas. Therefore, a statistical analysis using a large sample size is needed to fully explore the relationship between age and survival in patients with glioma. In the clinical cohort studies mentioned above, the population size of glioma patients was small, challenging the efficacy of statistical tests. Our study population included 66,465 glioma patients with complete clinical follow-up data. The mean follow-up for the total population was 24 months. This enables us to fully analyze the influence of age on the prognosis of patients with glioma using different multivariate models to adjust the influencing factors of covariates. Notably, our study observed that age 23 is an inflection point for glioma prognosis. There are different opinions on the relationship between age and glioma prognosis; however, most studies only transform age into a categorical variable for analysis, thus losing the advantage of a continuous variable. For the first time, we used restricted cubic spline plots to reveal the outcome of age as a continuous variable at different stages, possibly challenging the age restratification of gliomas.

The heterogeneity of the association between glioma age and prognosis in previous studies may have been attributed to the cutoff point differences in the age of glioma patients in published studies. This traditional analysis method converts age into dichotomous or multivariate variables in prediction models, such as univariate and multivariate regression analysis. This inevitably results in the loss of important information on continuity variables. In this case, the influence of different age classifications on the results was not compared, and the prediction efficiency of different models was not compared; therefore, the stability of the model was challenged. In our study, age, and survival risk ratios of patients with glioma were additionally examined using an adjusted multivariate regression model with RCS. The RCS function can provide a more accurate relationship between glioma patient prognosis and age change without changing the independent variables (continuous to categorical variables) [19]. Our analysis objectively illustrates the trend in the non-linear relationship between age and prognosis in patients with glioma, with two turning points in the younger age group (10 years and 23 years). The trend persisted after adjustment for covariates and was confirmed using the robustness of the internal validation analysis model and the age-prognosis relationship.

Our results challenge the current stratification method of changing age into a classification variable as a predictor of glioma prognosis. Various age cutoff points are being spread among people, and these data suggest that the mortality rate of patients with glioma increases dramatically with age [18, 20]. However, it should be emphasized that the results of previous studies are mostly based on small sample size and lack of adjustment of covariates. Therefore, it is important to reevaluate the relationship between age and glioma prognosis, and using age as a classification variable seems too arbitrary. Furthermore, different research results depend on the age of the segmentation point, indicating that the current age classification model is ineffective for the prognosis and survival of glioma, which may affect the individual treatment decisions of patients of different ages.

The results of our multivariate Cox regression study were not significant (1–9, 10–19, 20–29 years); however, two inflection points in age (10 and 23 years) were observed in the RCS plot. Histological stratification revealed that this trend persisted in HGG. Furthermore, the relationship between increased glioma age and mortality is not entirely clear; however, studies have observed that the expression of biomarkers in patients' cells changes with age. Batchelor et al. revealed that age influenced the prognosis of patients with glioma by influencing TP53, 1p, CDKN2A/p16 [21]. In addition, H3K27M mutation of other biomarkers and high expression of Ki67 and IDH1 wild-type glioma are related to the age of patients, thus affecting the survival probability [22–25]. Krigers et al. analyzed the outcomes of 99 patients with WHO 2 and 3 grade diffuse and anaplastic glioma using neuropathology and radiology and observed that age was an independent factor of prognosis only when IDH1 was wild-type [26]. Therefore, it is necessary for future research to conduct subgroup analysis of molecular characteristics of different gliomas to provide personalized treatment options.

There were some limitations in our study. First, the limitations of the SEER public database. SEER database has the advantage of a large tumor sample size; however, it still has some disadvantages, such as data coding errors and glioma recurrence information not being collected. Second, retrospective studies may be biased in population selection. Despite these limitations, our study had the advantage of analyzing clinical data from a large sample of glioma patients. The model constructed by adjusting covariates according to existing data has high accuracy. Furthermore, we used RCS smoothing curves to reveal the relationship between patient age and prognosis without setting reference groups in advance and excluded the statistical effect of age transformation into a categorical variable. This objective and rigorous statistical approach evaluates the relationship between continuous variables and verifies the reliability of the results with the results of internally validated statistical techniques.

## Conclusions

Our large sample size study provides valuable information for clinical reference based on the relationship between age and prognosis of patients with glioma. It has been widely introduced that the age of glioma patients is an important prognostic factor by grouping them into children, adults, and the elderly. Current studies have revealed that age is not completely positively correlated with prognosis. There were two inflection points at 10 and 23 years of age, and there was a J-shaped correlation between age and prognosis after 23 years of age. These results adjust the applicability of current age groupings and suggest the inadequacy of converting continuous variables into categorical variables. The results of this study are timely and necessary because they involve the development of future models of age and prognosis for gliomas, including glioblastoma. The current age grouping model for glioma may be reconsidered to explore a more accurate risk prediction model to guide the individualized treatment of patients with glioma at different ages.

## Supplementary Information

**Additional file 1.** 

## Abbreviations

CNS: central nervous system
GBM: glioblastoma multiforme
WHO: World Health Organization
LGG: low grade gliomas
HGG: high grade gliomas
SEER: Surveillance, Epidemiology, and End Results
ICD-O-3: International Classification Diseases for Oncology, third edition
RCS: restricted cubic spline
AIC: Akaike information criterion
