# Supplementary figures and images for: Exploring the relationship between age and prognosis in glioma: rethinking current age stratification
Source: BMC Neurol. 2022 Sep 15;22:350. doi: 10.1186/s12883-022-02879-9 (PMC9476578; doi:10.1186/s12883-022-02879-9)

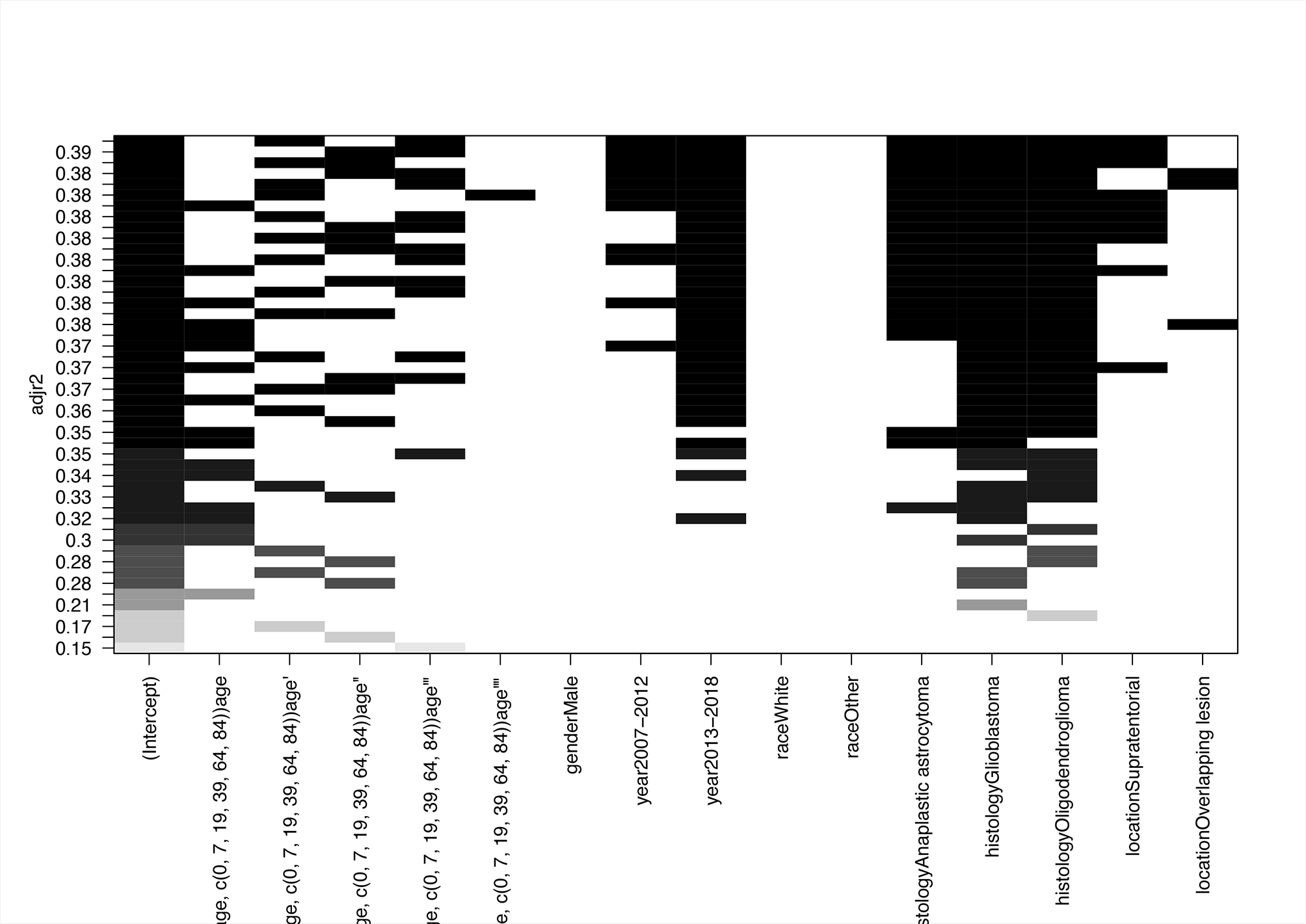


Figure legend

supplementary figure 1: Best subsets regression result.

Supplement: Supplementary file 1 — Additional file 1. [file 12883_2022_2879_MOESM1_ESM.docx]
